# Supplementary material for: Complexome profiling of the Chlamydomonas psb28 mutant reveals TEF5 as an early PSII assembly factor
Source: Plant Cell. 2025 Mar 17;37(6):koaf055. doi: 10.1093/plcell/koaf055 (PMC12164590; doi:10.1093/plcell/koaf055)
Supplement: koaf055_Supplementary_Data [file koaf055_supplementary_data.zip › psb28_interactive.zip!/plots/intensity/qualityPlots/peptides/TIM21_Cre09.g405550.t1.2.html]

### Cre09.g405550.t1.2

Trivial name: TIM21  
SeqCoverage [median]: 19.60  
Mass: 29.20 kDa  
  
 

| Sequence | MissedCleavage | Mass [Da] | wtIntensity | muIntensity | Score |
| --- | --- | --- | --- | --- | --- |
| AWGSNSESR | 0 | 992.43 | 243.54 | 0.00 | 52.48 |
| DKYDSLELTQER | 1 | 1495.72 | 0.00 | 1962.79 | 60.63 |
| IDSITDK | 0 | 790.41 | 358.73 | 671.70 | 87.32 |
| IGEAGDIR | 0 | 829.43 | 5067.46 | 11331.52 | 109.01 |
| LTIVSPPR | 0 | 881.53 | 2210.71 | 5125.54 | 126.28 |
| QQIPHQIYK | 0 | 1153.62 | 0.00 | 319.37 | 51.85 |
|  |  |  | 7880.4 | 19410.9 |  |
